# Characteristics and outcomes for hip fracture patients in an integrated orthogeriatric care model: a descriptive study of four discharge pathways with one-year follow-up

**DOI:** 10.1186/s12891-025-08427-z

**Published:** 2025-02-24

**Authors:** Eirik Solheim Salvesen, Kristin Taraldsen, Greger Lønne, Stian Lydersen, Sarah Elizabeth Lamb, Kjersti Opdal, Ingvild Saltvedt, Lars Gunnar Johnsen

**Affiliations:** 1 Department of Neuromedicine and Movement Science, NTNU, Trondheim, Norway; 2 Department of Orthopaedic Surgery, Sørlandet Hospital HF, Arendal, Norway; 3 Department of Rehabilitation Science and Health Technology, OsloMet, Oslo Norway; 4 Department of Orthopaedic Surgery, Innlandet Hospital HF, Lillehammer, Norway; 5 The Regional Centre for Child and Youth, Department of Mental Health, NTNU, Trondheim, Norway; 6 Faculty of Health and Life Sciences, University of Exeter, Exeter, UK; 7 Department of Geriatrics, St Olav`s Hospital HF, Trondheim, Norway; 8 Department of Orthopaedic Surgery, St Olav`s Hospital HF, Trondheim, Norway

**Keywords:** Hip fracture, Differentiated treatment, Discharge pathway, Pre-fracture function, Integrated orthogeriatric care

## Abstract

**Background:**

Orthogeriatric hospital care is recommended for hip fra cture patients, but differentiated hospital care has not been evaluated. The aim of this study was to describe physical performance and health-related quality of life for hip fracture patients 1-year after surgery in four treatment pathways. We also report changes in functional outcomes from baseline to 1-year follow-up together with readmission and mortality rates for each pathway.

**Methods:**

We included 177 hip fracture patients aged 65 years or older from a single center in Norway. Participants were discharged home, to specialised rehabilitation, regular rehabilitation or nursing home based on orthogeriatric assessment of pre- and postfracture function, mobility level and Ac tivities of Daily Living. Outcome variables included Short Physical Performance Battery, EuroQol-5-dimension-5-level, Barthel-index, Lawton & Brody Instrumental Activities of Daily Living, Lawton & Brody Self-Maintenance Scale, readmission and mortality rates during follow-up.

**Results:**

Participants discharged home and to specialised rehabilitation were younger and healthier than participants discharged to regular rehabilitation and nursing home. All groups had a clinically important improvement in Short Physical Performance Battery score (mean 4.8 points, 95% confidence interval (CI) 4.2, 5.5) from post-surgery to 1-year follow-up and a clinically important decline in EuroQol-5-dimension-5-level (mean -0.12 points, CI -0.16, -0.07) from baseline to 1-year follow-up. The decline in Barthel-index from baseline to 1-year follow-up was greater in the regular rehabilitation group (mean –2.3 points, CI -4.2, -0.2) than in the home group (mean -0.6 points, CI -1.4, 0.2) and specialised rehabilitation group (mean -0.4 points, CI -2.4, 1.6). Participants in the regular rehabilitation group were more frequently readmitted (standardised Pearson residual 4.1) and mortality rates were higher in the nursing home group (standardised Pearson residual 7.8) during the first year.

**Conclusions:**

Orthogeriatric treatment pathways for hip fracture patients entailed differentiation based on factors such as age, mobility, comorbidity and physical function. Participants in all pathways improved in physical performance-scores, yet experienced decline in quality of life-scores during follow-up. Overall readmission and mortality rates were not influenced, but varied between pathways. Further research is needed to investigate the need for differentiated hospital treatment and its potential effects on rehabilitation after discharge.

## Introduction

Orthogeriatric care entails systematic collaboration between orthopaedic surgeons and geriatricians through multi- or interdisciplinary teams in treatment of older adults with fractures [1]. Orthogeriatric treatment can be organised differently [2], and treatment models varies from ad hoc geriatric consultation in an orthopaedic ward to shared care in an orthogeriatric unit [3]. Interdisciplinary orthogeriatric care with early mobilisation is shown to prevent loss of function after hip fractures [1, 2, 4, 5] and improve mobility, cognition and ability to perform activities of daily living (ADL) [6]. Implementing clinical orthogeriatric treatment pathways has been associated with lower 30-day mortality [7] and better physical performance [4]. However, there are no beneficial effects on quality of life [7] compared to usual orthopaedic care [4, 8]. Still, guidelines recommend interdisciplinary orthogeriatric treatment to optimise outcomes [1, 2, 5, 9].

Most hip fracture patients are older adults and frail [10] with multiple comorbidities and polypharmacy [11, 12]. These patients often have increased morbidity and mortality regardless of hospital treatment [12] with reduced ability to perform ADL after surgery [2, 13]. However, hip fracture patients are a heterogeneous group even before their fracture [14–17]. Factors such as younger age, intracapsular fracture type and high pre-fracture functional status are associated with better outcomes after surgery [18]. Thus, studies have suggested clustering hip fracture patients into more homogenous groups and recommended different care pathways to better predict outcomes and target interventions [14–17]. Differentiated treatment pathways may streamline in-hospital treatment and better allocate hospital resources. To our knowledge, differentiated treatment pathways have not been used with orthogeriatric care models.

In March 2019, an orthogeriatric care model was established at St. Olav`s Hospital, Trondheim, Norway. Four treatment pathways were created as a collaborative effort between the hospital and the health care services from two nearby municipalities. We hypothesised that treatment through differentiated pathways could facilitate patient-centred rehabilitation during and after hospitalisation. This study aims to report the physical performance and health-related quality of life for patients across four treatment pathways one year after surgery. We also aim to describe changes in functional outcomes from baseline to 1-year follow-up and readmission and mortality rates for patients in each pathway.

## Methods

### Study design

This prospective cohort study included hip fracture patients admitted to the orthopaedic department at St. Olav`s Hospital in Trondheim, Norway.

### Inclusion criteria

Participants were patients aged 65 or older with an acute hip fracture after low-energy trauma residing in Trondheim (200 000 inhabitants) and Melhus (17 000 inhabitants) communities. Only the first fracture episode was included if patients were hospitalised more than once. We excluded patients with pathological fractures, periprosthetic fractures and patients not able to understand the Norwegian language.

#### Setting

St. Olav`s Hospital, the University Hospital of Trondheim, serves a population of approximately 300,000 and treats approximately 400 hip fractures per year. Until 2018 hip fracture patients were offered standard orthopaedic care with geriatric consultation on request. From 2018 a restructuring of fracture care was undertaken, and an integrated orthogeriatric unit with shared care [1–3] was created in the orthopaedic department. From March 24th 2019 to April 30th 2021 most hip fracture patients above 65 years admitted in the orthopaedic traumatology ward at St. Olav`s Hospital were treated in this orthogeriatric unit. All patients received orthogeriatric assessment, medical evaluation and physiotherapy during hospital stay. All participants were postoperatively allowed immediate weight-bearing as tolerated. The treatment pathways used in this study were created in collaboration with one specialised rehabilitation center and two nearby municipalities (Trondheim and Melhus), and these two municipalities were considered prepared for reception of dischargeable patients. We therefore only included patients from Trondheim and Melhus in this study. The orthogeriatric care model is further described in Appendix 1.

#### Treatment pathways and patient groups

Participants were preoperatively labelled to a tentative pathway based on prehospital residence, preoperative ADL function and mobility level. The participants were finally categorised into one of four treatment pathways on day 1 or 2 after surgery. The selection was based on orthogeriatric assessments including clinical evaluations of pre- and postoperative ADL function, cognition, frailty, preoperative mobility level and postoperative physical performance, and motivation of patient and caregivers. The decisions were collectively made by the interdisciplinary team members through daily collaboration meetings. Due to poor capacity in the interdisciplinary team, the orthogeriatric work-up and early rehabilitation had to be prioritised and differentiated based on patient characteristics. Home-dwelling participants with high ADL function and mobility were discharged home with or without health care assistance (pathway 1). They received physiotherapy twice daily and evening mobilisation with nurses during hospitalisation. The rehabilitation after discharge was given through municipal response teams including physiotherapists and home care nursing or personal physiotherapy sessions. Home-dwelling participants with high pre-fracture function and reduced post-fracture function, making discharge directly home impossible, were discharged to specialised rehabilitation (pathway 2). They received the same intensity of physiotherapy and evening mobilisation as participants in pathway 1. The rehabilitation program after discharge in pathway 2 was organised as a department of the hospital and mimicked the rehabilitation given in the orthopaedic ward, and is therefore referred to as specialised rehabilitation. The specialised rehabilitation centre accepted patients for maximum two weeks of rehabilitation before patients were discharged home. Frail, home-dwelling participants with low ADL function and greater need of health care assistance after surgery were prioritised for a short term rehabilitation stay that was expected to last up to four weeks in community rehabilitation centres (pathway 3). They received physiotherapy, but had more arbitrary evening mobilisation than participants in pathway 1 and 2 during hospitalisation. We have no information on the rehabilitation given to participants in pathway 3 after discharge, but have called it regular rehabilitation since this is the standard rehabilitation pathway for patients not able to go home in Norway. Home-dwelling participants who were very frail with very low ADL function prior to and after fracture and participants admitted from nursing homes were discharged to permanent residence in a community nursing home after medical stabilisation in the hospital (pathway 4). Although these participants were offered physiotherapy during hospital stay, they were often unable to make use of it. We have no information on the rehabilitation given after discharge in pathway 4. Only participants in pathways 1 and 2 were evaluated by an occupational therapist. The pathways are also described in Appendix 1.

### Data collection

At hospital admission date (baseline), patients were asked to fill out questionnaires to assess their quality of life and pre-fracture ADL function. Additional data, including time from admission to surgery, surgical treatment, length of stay and postoperative medical complications were consecutively registered during hospital stay. Patients were offered a telephone interview with a research nurse 3 months postoperatively answering the EuroQol-5-Dimentions-5-Level-questionnaire (EQ-5D-5L). All patients were also offered a physical examination by a physiotherapist and X-ray of the operated hip 1 year after surgery. Information on readmissions and mortality rates was obtained from hospital medical records.

#### Measurements

Background data, including gender, age, Body Mass Index (BMI), living condition, pre-fracture mobility, fracture type and comorbidity were registered at hospitalisation. Comorbidity was assessed using the Charlson comorbidity index (CCI) [19, 20] and the American Society of Anaesthesiologists (ASA) score [21]. We utilised a version with 12 items and a sum score ranging from 0 to 24 for CCI [22]. A high score indicates greater mortality risk and more severe comorbid conditions [20]. The ASA score was decided by the anaesthesiologist before surgery. This score ranges from 1 to 5, where a high score indicates higher morbidity and greater mortality risk [21].

We utilised the Short Physical Performance Battery (SPPB) to evaluate physical performance. The SPPB [23] consist of three tests, each scored from 0 to 4, resulting in a total score ranging from 0 to 12 [24]. Lower scores indicate significant functional impairment [23]. A change of 0.5 points is considered a minimal clinically important difference (MCID) [24, 25]. The SPPB was assessed by a physiotherapist as close to discharge as possible and at 1-year follow-up.

Health-related quality of life (HRQoL) was assessed with the EuroQol-5-Dimentions-5-Level (EQ-5D-5L) [24, 26]. We transformed the answers on EQ-5D-5L into an index score using a crosswalk-algorithm from UK EQ-5D-3L to EQ-5D-5L with Norwegian population value sets [27]. The EQ-5D-5L index ranges from − 0.285 (worst imaginable health state) to 1 (perfect health) [24]. A 0.08-point change is considered MCID [24, 28]. The EQ-5D-5L index was assessed at hospitalisation, during telephone interview 3-months postoperatively and at 1-year follow-up. Participants were assisted by their next of kin when or if needed to recall their HRQoL a week before the fracture and follow-up dates.

The modified Barthel-index-20 [29] and Lawton & Brody scales [30] were used to evaluate ADL function. The Barthel-index-20 consists of 10 items with a total score from 0 to 20; a high score indicates a better ability to perform ADL [29]. A change of 2.0 points is considered MCID [31]. Lawton & Brody Instrumental ADL (I-ADL) has 8 items and ranges from 0 to 8 for women and 0 to 5 for men [30]. A change of 0.5 points is considered MCID [32]. Lawton & Brody Physical Self-Maintenance Scale (PSMS), which measures basic ADL, has 6 items with a total score from 0 to 6 [30]. Higher scores in the Lawton & Brody forms indicate higher levels of ADL function [30, 33]. Scores were evaluated at hospitalisation and at 1-year follow-up. Participants were assisted by their next of kin when or if needed to recall their ADL function a week before the fracture and follow-up dates. The Barthel-index was also assessed on day 3–5 after surgery in the hospital.

Primary outcome measures per patient pathway included the SPPB score and EQ-5D-5L index at 1-year follow-up. Secondary outcome measures were ADL-scores, readmission and mortality rates during follow-up.

#### Statistical analysis

Continuous data are reported as mean and standard deviation (SD), categorical data as counts and percentages. Proportions were compared using the Pearson chisquared test. Standardized Pearson residuals exceeding an absolute value of 2 were considered indications of significantly deviating proportions [34]. We used linear mixed models with Barthel-index, Lawton & Brody scores and SPPB scores, one at a time, as dependent variables to analyse the changes in these scores from baseline to follow-up. We included the pathways, time and their interactions as covariates and the patient as random effect. The Lawton & Brody Instrumental ADL score was analysed for men and women separately. Changes in EQ-5D-5L index were analysed with a similar mixed linear model. The nursing home pathway was omitted from pathway analyses due to low patient recruitment. Normality of residuals were confirmed by visual inspection of Q-Q-plots. We considered p-values below 0.05 statistically significant and report 95% confidence intervals (CI) when relevant. Statistical analyses were performed with IBM SPSS Statistics (version 29.0, IBM Corp. Armonk, New York).

## Results

**Fig. 1:**
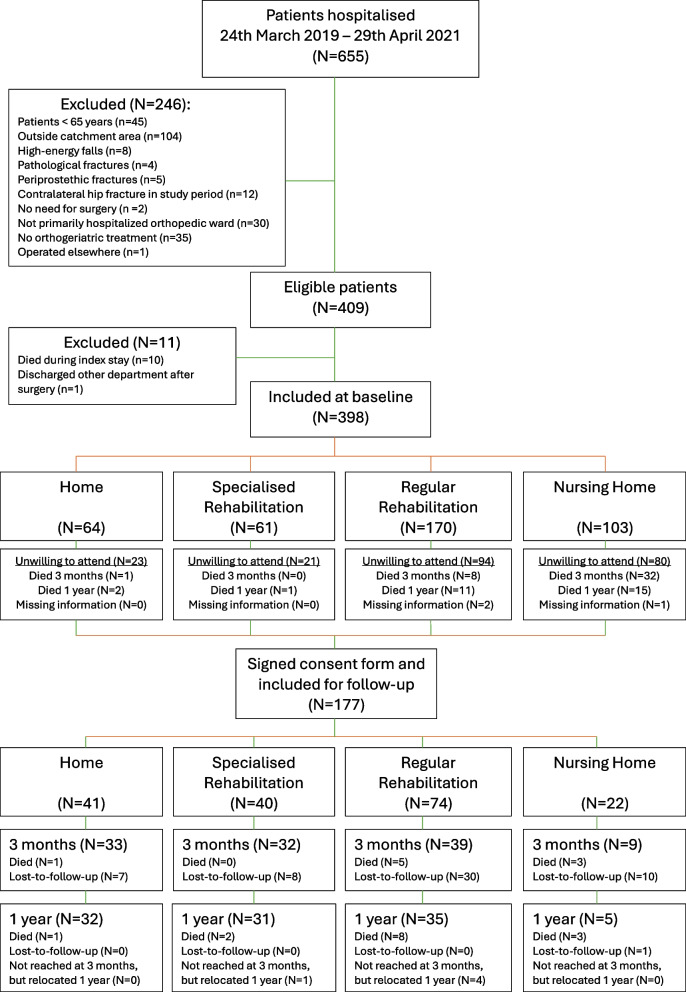
Flowchart

We included 409 patients in the study of 30-day, 90-day, and 1-year mortality rates. As ten patients died during hospital stay, and one patient did not receive postoperative treatment in the orthopaedic department, we included 398 participants with baseline data. In total 177 (44.5%) consented to participate the 1-year follow-up. Figure 1 illustrates the patient flow chart.

### Baseline characteristics

Demographics are shown in Table 1. Participants mean age was 83.0 years (SD 8.5), and 70.9% were women. There were 58.5% femoral neck fractures, 39.4% pertrochanteric fractures and 2.0% subtrochanteric fractures. Participants discharged home (*n* = 64, mean age 78.3, SD 9.4) and to specialised rehabilitation (*n* = 61, mean age 78.8, SD 7.0) were younger than participants discharged to regular rehabilitation (*n* = 170, mean age 84.5, SD 8.2) and nursing home (*n* = 103, mean age 86.0, SD 7.0). Participants in the nursing home group had a higher CCI (mean 3.0, SD 1.7) than other pathways. The ASA scores were lower in the home group (mean 2.6, SD 0.6) and specialised rehabilitation group (mean 2.6, SD 0.6) compared to the regular rehabilitation gr oup (mean 3.0, SD 0.6) and nursing home group (mean 3.2, SD 0.5). More participants in the nursing home group (*n* = 89, 86.4%) had been diagnosed with Dementia before admission compared with the home group (*n* = 4, 6.2%), specialised rehabilitation group (*n* = 0) and regular rehabilitation group (*n* = 27, 16.1%).

**Table 1:** Baseline characteristics

**Included, N (%)**	**Total** **N = 398**	**Home** **N = 64 (16.1)**	**Specialised rehabilitation** **N = 61 (15.3)**	**Regular rehabilitation** **N = 170 (42.7)**	**Nursing home** **N = 103 (25.9)**
**Age at hospitalization (years), **					
Mean (SD)	83.0 (8.5)	78.3 (9.4)	78.8 (7.0)	84.5 (8.2)	86.0 (7.0)
**Charlsons comorbidity index (0-24), **					
Mean (SD)	1.8 (1.9)	1.2 (1.5)	1.4 (2.0)	1.5 (1.9)	3.0 (1.7)
**Gender**					
- Women,N (%)	282 (70.9)	43 (67.2)	48 (78.7)	113 (66.5)	78 (75.7)
**Admitted from **					
- Home, N (%)	258 (64.8)	49 (76.5)	60 (98.3)	144 (84.7)	5 (4.8)
- Care home, N (%)	27 (6.8)	13 (20.3)	0	10 (5.9)	4 (3.9)
- Rehabilitation stay , N (%)	5 (1.2)	0	1 (1.7)	3 (1.7)	1 (1.0)
- Nursing home, N (%)	67 (16.8)	0	0	2 (1.2)	65 (63.1)
- Not registered, N (%)	41 (10.3)	2 (3.1)	0	11 (6.4)	28 (27.2)
**Mobility before fracture**					
- Without aid, N (%)	201 (50.5)	44 (68.7)	52 (85.2)	86 (50.6)	19 (18.4)
- Walking-aid, N (%)	188 (47.2)	16 (25.0)	9 (14.7)	81 (47.6)	82 (79.6)
- Wheel chair, N (%)	9 (2.2)	4 (6.2)	0	3 (1.7)	2 (1.9)
**Fracture type**					
- Femoral neck (FCF), N (%)	233 (58.5)	49 (76.5)	34 (55.7)	95 (55.9)	55 (53.4)
- Pertrochanteric, N (%)	157 (39.4)	13 (20.3)	25 (41.0)	72 (42.3)	47 (45.6)
- Subtrochanteric, N (%)	8 (2.0)	2 (3.1)	2 (3.3)	3 (1.7)	1 (0.9)
**Surgical treatment**					
- Screws, N (%)	40 (10.0)	14 (21.8)	4 (6.5)	11 (6.5)	11 (10.7)
- Bipolar hemiarthroplasty, N (%)	168 (42.2)	19 (29.7)	25 (41.0)	82 (48.2)	42 (40.8)
- Total hip replacement, N (%)	22 (5.5)	14 (21.8)	4 (6.5)	3 (1.7)	1 (0.9)
- Bone plates and screws, N (%)	89 (22.3)	8 (12.5)	15 (24.6)	38 (22.3)	28 (27.2)
- Intramedullary nail, N (%)	79 (19.8)	9 (14.1)	13 (21.3)	36 (21.2)	21 (20.4)
**ASA (1-5) **					
- 1, N (%)	5 (1.2)	2 (3.1)	0	3 (1.7)	0
- 2, N (%)	81 (20.3)	24 (37.5)	28 (45.9)	25 (14.7)	4 (3.8)
- 3, N (%)	253 (63.5)	36 (56.2)	30 (49.2)	117 (68.8)	70 (67.9)
- 4, N (%)	58 (14.6)	2 (3.1)	3 (4.9)	25 (14.7)	28 (27.2)
- 5, N (%)	1 (0.2)	0	0	0	1 (0.9)
**Previous diagnoses**					
- Dementia, N (%)	120 (30.1)	4 (6.2)	0	27 (16.1)	89 (86.4)
- Diabetes, N (%)	64 (16.2)	11 (17.2)	4 (6.5)	31 (18.5)	18 (17.5)
- Cancer, N (%)	94 (23.8)	10 (15.6)	18 (29.5)	42 (25.1)	24 (23.3)
- Kidney disease, N (%)	31 (7.8)	4 (6.2)	1 (1.6)	16 (9.6)	9 (5.4)
- Heart disease, N (%)	250 (63.3)	29 (45.3)	32 (52.4)	107 (64.1)	82 (49.1)
- Lung disease, N (%)	72 (18.2)	18 (28.1)	13 (21.3)	23 (13.8)	18 (10.8)

### Performance-based test after surgery

We observed a higher SPPB score in the home group (mean 4.1, SD 2.5) after surgery compared to the specialised rehabilitation group (mean 1.9, SD 1.6) and regular rehabilitation group (mean 0.8, SD 1.4).

### Patient Reported Outcome Measures (PROMs) at baseline

The regular rehabilitation group had lower EQ-5D-5L index (mean 0.74, SD 0.21) compared to the home group (mean 0.87, SD 0.17) and specialised rehabilitation group (mean 0.86, SD 0.16). The regular rehabilitation group had lower scores for Lawton & Brody Instrumental ADL for women (mean 6.2, SD 1.8) compared to the home group (mean 7.6, SD 0.8) and specialised rehabilitation group (mean 7.6, SD 0.7), and lower scores for Physical Self-Maintenance Scale (mean 4.6, SD 1.3) than the home group (mean 5.3, SD 1.1) and specialised rehabilitation group (mean 5.3, SD 0.7).

### Performance-based test at 1-year follow-up

All groups had an improvement in SPPB scores (mean 4.8, CI 4.2, 5.5) from post-surgery to 1-year follow-up. The change in SPPB score was 1.7 points larger in the specialised rehabilitation group (5.4, CI 2.7, 8.1) compared to the home group (3.7, CI 2.6, 4.8).

### Patient Reported Outcome Measures (PROMs) at 1-year follow-up

All groups had a clinically meaningful decline in HRQoL (−0.12, CI −0.16, −0.07) from baseline to 1-year follow-up. The decline in Barthel-index at 1-year follow-up was greater in the regular rehabilitation group (−2.3, CI −4.2, −0.2) compared with the home group (−0.6, CI −1.4, 0.2) and specialised rehabilitation group (−0.4, CI −2.4, 1.6).

**Fig. 2:**
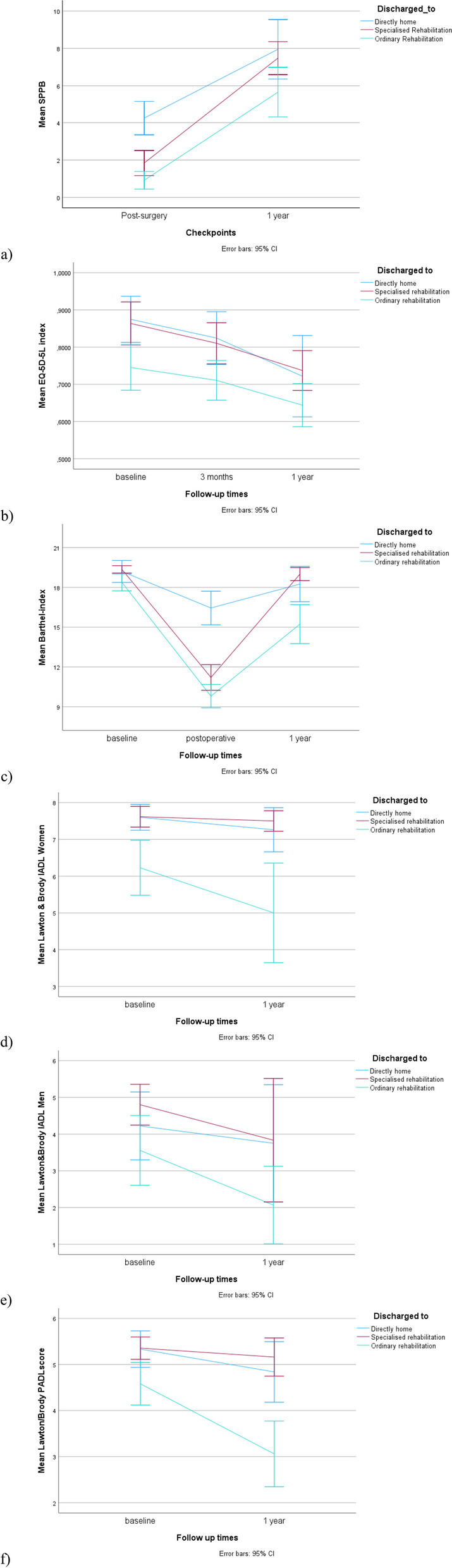
Graphic illustrations of the change in physical performance, ADL- and quality of life-scores during the first year

SPPB after surgery and 1-year follow-up and PROMs at baseline and 1-year follow-up for included participants are shown in Table 2. The change from post-surgery to 1-year follow-up for SPPB and from baseline to 1-year follow-up for each PROM are illustrated in Fig. 2 a-f.

**Table 2:** Physical performance, adl-function and q uality of life

	**TotalN = 177**		**HomeN = 41**		**Specialised rehabilitationN = 40**		**Regular rehabilitationN = 74**		**Nursing homeN = 22**	
	**N**	**Mean (SD)**	**N**	**Mean (SD)**	**N**	**Mean (SD)**	**N**	**Mean (SD)**	**N**	**Mean (SD)**
**Short Physical Performance Battery (SPPB) (0-12)**										
Post-surgery^b^	118	2.0 (2.3)	34	4.1 (2.5)	28	1.9 (1.6)	46	0.8 (1.4)	10	0.3 (0.9)
1 year after treatment	70	7.0 (3.0)	22	7.9 (3.6)	27	7.5 (2.2)	20	5.6 (2.8)	1	3.0 (^a^)
Change^c^, mean (95% CI)		4.8 (4.2, 5.5)		3.7 (2.6, 4.8)		5.4 (2.7, 8.1)		4.9 (2.2, 7.6)		2.7(^d^)
**EQ-5D-5L index (-0.285-1)**										
Baseline	120	0.80 (0.21)	34	0.87 (0.17)	33	0.86 (0.16)	49	0.74 (0.21)	4	0.42 (0.22)
3 months after treatment	112	0.75 (0.19)	33	0.82 (0.20)	31	0.81 (0.15)	39	0.71 (0.16)	9	0.50 (0.16)
1 year after treatment	92	0.68 (0.23)	30	0.72 (0.29)	31	0.73 (0.14)	28	0.64 (0.15)	3	0.23 (0.41)
Change^c^, mean (95% CI)		-0.12 (-0.16, -0.07)		-0.13 (-0.20, -0.05)		-0.13 (-0.31, 0.05)		-0.11 (-0.29, 0.07)		-0.19 (-0.51, 0.13)
**Barthel-index (0-20)**										
Baseline	94	18.9 (1.7)	30	19.2 (2.2)	31	19.3 (0.8)	32	18.4 (1.7)	1	12.7 (^a^)
Post-surgery^b^	82	12.4 (3.9)	27	16.4 (3.2)	24	11.2 (2.2)	30	9.8 (2.3)	1	4.6 (^a^)
1 year after treatment	103	17.1 (4.0)	32	18.3 (3.7)	31	19.0 (1.3)	35	15.2 (4.2)	5	11.4 (5.0)
Change^c^, mean (95% CI)		-1.3 (-2.0, -0.6)		-0.6 (-1.4, 0.2)		-0.4 (-2.4, 1.6)		-2.3 (-4.2, -0.2)		-1.5 (^d^)
**Lawton&Brody I-ADL women (0-8)**										
Baseline	69	7.1 (1.3)	20	7.6 (0.8)	26	7.6 (0.7)	22	6.2 (1.8)	1	2.6 (^a^)
1 year after treatment	71	6.4 (2.3)	23	7.3 (1.4)	24	7.5 (0.7)	19	5.0 (2.8)	5	2.2 (1.6)
Change^c^, mean (95% CI)		-0.4 (-0.7, -0.1)		-0.3 (-0.8, 0.1)		-0.1 (-1.3, 1.1)		-0.8 (-2.0, 1.1)		-0.4(^d^)
**Lawton&Brody I-ADL men (0-5)**										
Baseline	23	4.1 (1.2)	9	4.2 (1.2)	5	4.8 (0.5)	9	3.5 (1.2)	0	Not applicable
1 year after treatment	29	2.9 (2.0)	8	3.7 (1.9)	6	3.8 (1.6)	15	2.1 (1.9)	0	Not applicable
Change^c^, mean (95% CI)		-0.5 (-1.0, -0.1)		-0.1 (-0.8, 0.6)		-0.8 (-2.6, 1.0)		-0.8 (-2.6, 1.0)		Not applicable
**Lawton&Brody Physical ADL (PSMS) (0-6)**										
Baseline	93	5.1 (1.0)	30	5.3 (1.1)	31	5.3 (0.7)	31	4.6 (1.3)	1	3.2 (^a^)
1 year after treatment	101	4.2 (2.0)	31	4.8 (1.8)	31	5.2 (1.1)	34	3.1 (2.0)	5	1.6 (2.2)
Change^c^, mean (95% CI)	101	-0.5 (-0.7, -0.2)		-0.4 (-0.9, 0.1)		-0.2 (-1.2, 0.9)		-0.9 (-1.9, 0.3)		-1.6(^d^)

The numbers from each follow-up time are derived from descriptive statistics. The change from baseline to 1-year was calculated using linear mixed models

^a^No standard deviation due to one observation

^b^Post-surgery Barthel-index and SPPB were measured as close to discharge as possible, between day 3-5 after surgery

^c^Change from baseline to 1-year follow-up

^d^The number of observations were too low to compute informative confidence intervals

### Hospital data

Hospital characteristics are shown in Table 3. Participants discharged to nursing home had shorter hospital stays (mean 4.0 days, SD 2.1) and participants discharged to regular rehabilitation had longer hospital stays (mean 8.6, SD 4.7) than the three other groups. Mean time to surgery was 24.1 h (SD 18.1), and no clinically relevant difference in mean time to surgery was observed between the four groups. Delirium was the most frequent complication (*n* = 148, 37.2%) during hospital stay, followed by blood transfusions (*n* = 138, 34.6%) and urinary tract infections (*n* = 48, 12.1%).

**Table 3:** Hospital characteristics and mortality

**Hospital Characteristics**	**Total** **N = 398**	**Home** **N = 64**	**Specialised rehabilitation** **N = 61**	**Regular Rehabilitation** **N = 170**	**Nursing home** **N = 103**
**Index stay (days), **					
- Mean (SD)	6.5 (4.1)	5.9 (3.2)	5.2 (2.2)	8.6 (4.7)	4.0 (2.1)
**Time to surgery (hours), **					
- Mean (SD)	24.1 (18.2)	25.5 (16.9)	24.8 (15.7)	24.6 (20.7)	22.0 (15.7)
**Complications during index stay**					
- Blood transfusion, n (%)	138 (34.6)	13 (20.3)	18 (29.5)	65 (38.2)	42 (40.8)
- Delirium, n (%)	148 (37.2)	9 (14.0)	5 (8.2)	67 (39.4)	67 (65.0)
- Pneumonia, n (%)	35 (8.8)	4 (6.2)	4 (6.6)	20 (11.7)	7 (6.8)
- Urinary tract infection, n (%)	48 (12.1)	2 (3.1)	7 (11.5)	27 (15.9)	12 (11.6)
- Kidney failure, n (%)	40 (10.0)	4 (6.2)	3 (4.9)	19 (11.2)	14 (13.6)
- Reoperations, n (%)	4 (1.0)	0	0	4 (2.3)	0
**Readmission 3 months, n/surviving patients (%), Standardised Pearson residual**	**75/355**^**a**^ **(21.1)**	**10/62 (16.1), -1.1**	**7/61 (11.5), -2.0 **	**40/160**^**a**^ **(25.0), 1.6 **	**18/72**^**a**^ **(25.0), 0.9**
- Surgical complication, n (%)	13/75 (17.3)	0	1/7 (14.2)	7/40 (17.5)	5/18 (27.8)
- New illness, n (%)	44/75 (58.6)	7/10 (70.0)	4/7 (57.1)	24/40 (60.0)	9/18 (50.0)
- Other reasons, n (%)	18/75 (24.0)	3/10 (30.0)	2/7 (28.6)	9/40 (22.5)	4/18 (22.2)
**Readmission 1 year, n/surviving patients (%), Standardised Pearson residual**	**152/333**^a^** (45.6) **	**19/60**^**a**^ **(31.7), -2.4**	**21/60**^**a**^ **(36.2), -1.8 **	**88/152**^**a**^ **(57.9), 4.1**	**24/61**^**a**^ **(39.3), -1.1**
- Surgical complication, n (%)	16/152 (10.5)	3/19 (15.8)	3/21 (14.3)	6/88 (6.8)	4/24 (16.7)
- New illness, n (%)	103/152 (67.7)	13/19 (68.4)	13/21 (61.9)	66/88 (75.0)	11/24 (45.8)
- Other reasons, n (%)	34/152 (22.3)	4/19 (21.0)	5/21 (23.8)	16/88 (18.2)	9/24 (37.5)
**Mortality**	**Total** **N = 409**^**b**^	**Home** **N = 64**	**Specialised rehabilitation** **N = 61**	**Regular Rehabilitation** **N = 170**	**Nursing home** **N = 103**
**30-day mortality, n (%), Standardised Pearson residual**	**31 (7.5)**^b^	**1 (1.5), -1.5**	**0, -2.0**	**4 (2.3), -2.3**	**16 (15.5), 5.4**
In-hospital mortality, n (%)	**10 (2.4)**^c^				
**90-day mortality, n (%), Standardised Pearson residual**	**62 (15.1)**^b^	**2 (3.1), -2.5**	**0, - 3.2**	**14 (8.2), -2.6**	**35 (34.0), 7.6**
Died after discharge, other, n (%)	**1 (0.2)**^d^				
**1-year mortality, n (%), Standardised Pearson residual**	**104 (25.4)**^b^	**5 (7.8), -3.2**	**3 (4.9), -3.7**	**32 (18.8), -1.8**	**53 (51.4), 7.8**
Dead patients without pathway, n(%)	**11 (2.7)**				

^a^Four patients in the regular rehabilitation and nursing home group were both readmitted and died before 3 months (n=8). This also happened to 1 patient in the home group, 2 patients in the specialised rehabilitation group, 14 patients in the regular rehabilitation group and 11 patients in the nursing home group before 1 year follow-up (=28).

^b^All 409 eligible patients were included in the mortality analyses, including the eleven patients not allocated to a pathway.

^c^Ten patients died during hospital stay and before allocation to a pathway.

^d^One patient was transferred to another department postoperatively and was not given a treatment pathway.

### Readmission and mortality

The readmission rate was 21.1% at 3 months and 45.6% one year after hospitalisation (Table 3). There was no difference in readmissions between the groups at three months. Participants in the regular rehabilitation group were more frequently readmitted during the first year (standardised Pearson residual 4.1). A new illness was the most frequent reason for readmission at 3 months (*n* = 44, 58.6%) and 1 year (*n* = 103, 67.3%) in all groups.

The in-hospital mortality rate was 2.4% and the 30-day mortality rate was 7.5%. One year after hospitalisation 25.4% of participants had died. Patients in the nursing home group had a higher 30-day (standardised Pearson residual 5.4), 90-day (standardised Pearson residual 7.6) and 1 year (standardised Pearson residual 7.8) mortality than the three other pathways, and only 48.6% of patients in the nursing home group were alive one year after hospitalisation.

## Discussion

In the present study we included baseline data from 398 patients and follow-up results from 177 participants. Participants discharged home or to specialised rehabilitation were younger and healthier than participants discharged to regular rehabilitation or nursing home. All groups had an improvement in physical performance from post-surgery to 1-year follow-up and experienced a decline in self-reported quality of life from what they recalled having before the fracture to 1-year follow-up. Participants in the regular rehabilitation group were more frequently readmitted during the first year, and patients in the nursing home group had the highest mortality rate throughout the first year after surgery.

Implementing orthogeriatric care with treatment pathways entailed patient differentiation in our study. We found that participants discharged home or to specialised rehabilitation were younger and healthier than participants discharged to regular rehabilitation and nursing home. Previous studies have described similar discharge patterns [35–37]. One study found that hip fracture patients discharged home were healthier and had better pre-fracture function than patients discharged to acute or subacute rehabilitation facilities [37]. Another study showed that patients requiring rehabilitation in short-term nursing homes had poorer physical function than those discharged home [38]. The patient differentiation in our study was in line with previous findings on discharge patterns for hip fractures [35–38].

We found a higher increase in SPPB score from post-surgery to 1-year follow-up for participants in the specialised rehabilitation group compared with the home group and regular rehabilitation group. A review emphasised the importance of early mobilisation after surgery, and that physical activity and progressive resistance training during rehabilitation after discharge may improve patient`s mobility [39]. However, a recent study showed that a minority of patients have access to physiotherapy the first year after injury [40]. We neither have information on the frequency nor magnitude of physical therapy offered to participants in the four pathways after discharge. Therefore, we do not know if the observed variations in physical performance at 1-year follow-up in our study were incidental findings or due to differences in rehabilitation. Despite a great focus on optimising hospital treatment for geriatric hip fracture patients, little is known about what happens after discharge. It seems that the rehabilitation given after discharge have variability in magnitude, content and intensity, and some form of standardisation or even patient-centred rehabilitation could be warranted. Therefore, we believe knowledge of rehabilitation after hospitalisation should be a priority in future studies.

Previous studies on hip fracture patients have showed both an improvement [38] and a decline in health-related quality of life one year after surgery [7, 41]. In our study all treatment pathways showed a clinically relevant decline in EQ-5D-5L index from baseline to one year after hospitalisation. The pathways in our study differed in several factors known to affect EQ-5D-scores, such as age composition, pre-fracture mobility and comorbidity [42], but we found no difference in score change between the pathways during follow-up. This aligns well with a study from the Norwegian Hip Fracture Registry that found reduced quality of life for hip fracture patients in all age groups and for all fracture types [41] throughout the first year after hospitalisation. It may therefore be suggested that a hip fracture affects the self-reported quality of life for older adults in Norway regardless of hospital treatment.

We found better ADL scores at follow-up in the home group and specialised rehabilitation group compared to the regular rehabilitation group. Participants discharged to specialised rehabilitation nearly recovered their ADL function one year after surgery, whereas participants in the home group and regular rehabilitation group, respectively, had increasing degrees of decline in ADL scores from baseline to 1-year follow-up. Different trajectories of ADL scores for hip fracture patients have previously been described, where factors like low ASA score and younger age were linked to less decline in ADL level [12]. Our findings from the pathway analyses on ADL scores seem to resemble such trajectories [12]. Other studies have shown that functional dependence before fracture impacts recovery results after hip fractures [14, 16, 43, 44]. Even though we observed differences in prefracture self-reliance between our pathways, we were unable to show any impact of functional dependence on recovery results in this descriptive study.

Previous studies have reported no significant change in readmission rates with orthogeriatric care [1, 4] and 30-day readmission rates are reported to range between 10–15% [1]. Regrettably, we have not reported 30-day readmission in our study which makes direct comparison difficult. However, we found similar readmission rates at 3 months and 1 year as previously described for conventional orthopaedic care and fast-track care at our hospital [45] indicating that differentiated treatment in an orthogeriatric care model does not influence readmission rates at our facility. Interestingly, we found readmissions during follow-up to be more frequent among patients discharged to regular rehabilitation. This was not evident at 3 months, but significant 1 year after discharge. We do not know the reasons for these differences and can only speculate, but participants discharged to regular rehabilitation stayed longer in the hospital and we observed more reoperations and proportionally higher number of pneumonias during hospitalisation in this patient group. This could have increased patient vulnerability upon discharge. which may have delayed or influenced the rehabilitation given in the community centres after discharge. A previous study has stated that delayed rehabilitation could increase the risk for early readmission [46], but we have no data to confirm such a context in our study.

The 30-day and 1-year mortality rates in the present study were both lower [47, 48] and higher [49, 50] than comparable studies with orthogeriatric care models. Differentiated hospital treatment did not seem to influence neither 30-day nor 1-year mortality in our study. However, mortality was significantly higher for the older and more frail patients found in the nursing home group. This align well with what has been described by others [51].

### Strengths and limitations

One strength of this study was a pragmatic study design where participants were treated according to their functional status in a well-documented treatment model with patient-centred pathways. A pragmatic approach depicts the clinical everyday life and could show the unadorned results after the treatment of hip fracture patients. A pragmatic design also has some limitations, such as less control over cause and effect related to the treatment model compared to randomised controlled trials. Another limitation in our study was low recruitment of eligible patients, which is a known problem in studies of older adults with hip fractures. We were only able to recruit 44% of the eligible patients, and most of the patients who were unwilling to attend came from the regular rehabilitation and nursing home pathway. A third limitation was the missing follow-up data, and there was a high dropout rate among participants from regular rehabilitation. We do not know the reasons for this high dropout rate, but consider our data to be missing at random. However, we are aware of the possibility that participants with the best clinical outcomes are most likely to complete study assessments. Therefore, the high number of missing patients at recruitment and follow-up may have introduced confounding by selection in our study.

### Clinical implications

Health resources are scarce, and our multidisciplinary orthogeriatric model is resource-intensive. Although hospital treatment is more expensive with an orthogeriatric care model, it has been shown to save social expenses for permanent institutional places [6]. Differentiated treatment pathways with orthogeriatric care could streamline in-hospital treatment for hip fracture patients and thus facilitate patient-centred rehabilitation in the community. This study describes one attempt to streamline hospital treatment, but also depicts a knowledge gap on the content of post-discharge rehabilitation. We believe the latter needs to be further explored in order to clarify how post-discharge rehabilitation should be adapted in a patient-centred way.

## Conclusion

In this pragmatic study, we described the implementation of four treatment pathways in an orthogeriatric care model that differentiated participants based on age, pre-fracture and postoperative functions. All pathways had a clinically important improvement in SPPB score and a clinically important decline in EQ-5D-5L index at 1-year follow-up. Patients discharged to regular rehabilitation in community rehabilitation centers were more frequently readmitted during the first year. Patients discharged to nursing homes had higher mortality rates throughout 1-year follow-up. Further research is needed to evaluate if differentiated hospital treatment pathways facilitates patient-centred follow-up after discharge. The content of rehabilitation offered to geriatric hip fracture patients after discharge also needs to be addressed in future studies.

## Data Availability

The datasets used and/or analysed during the current study are available from the corresponding author on reasonable request.
